# The developmental emergence of differential brainstem serotonergic control of the sensory spinal cord

**DOI:** 10.1038/s41598-017-02509-2

**Published:** 2017-05-22

**Authors:** F. Schwaller, A. H. Kanellopoulos, M. Fitzgerald

**Affiliations:** 1 Department of Neuroscience, Physiology and Pharmacology, UCL, London, WC1E 6BT UK; 2 Department of Neuroscience, Max-Delbrück Centre for Molecular Medicine, Berlin-Buch, Germany

## Abstract

Descending connections from brainstem nuclei are known to exert powerful control of spinal nociception and pain behaviours in adult mammals. Here we present evidence that descending serotonergic fibres not only inhibit nociceptive activity, but also facilitate non-noxious tactile activity in the healthy adult rat spinal dorsal horn via activation of spinal 5-HT_3_ receptors (5-HT_3_Rs). We further show that this differential serotonergic control in the adult emerges from a non-modality selective system in young rats. Serotonergic fibres exert background 5-HT_3_R mediated facilitation of both tactile and nociceptive spinal activity in the first three postnatal weeks. Thus, differential descending serotonergic control of spinal touch and pain processing emerges in late postnatal life to allow flexible and context-dependent brain control of somatosensation.

## Introduction

The brain can powerfully modulate the processing of somatosensory information at lower levels of the central nervous system (CNS). Descending pathways from the periaqueductal grey (PAG) and the rostroventral medial medulla (RVM) inhibit and facilitate processing of somatosensory inputs to the spinal dorsal horn^1^. By increasing or decreasing the gain of spinal sensory processing, descending controls can modulate the output from the dorsal horn; either to action centres in the brain or to motor circuitry in the ventral spinal cord. Descending brainstem-spinal cord sensory controls are hypothesised to be one mechanism underlying endogenous pain controls such as placebo anaesthesia^2, 3^. There is considerable evidence that supraspinal pathways selectively target high-threshold nociceptive inputs in the spinal dorsal horn^4–7^, but earlier studies suggested that descending PAG-RVM control of spinal somatosensation is not nociceptive-selective and also targets non-noxious inputs^8, 9^. However, this evidence has been largely overlooked in recent studies.

Descending supraspinal modulation of spinal nociception is slow to mature in young rats. Descending PAG-RVM inhibition of noxious and C-fibre inputs in the dorsal horn is weak in the first postnatal weeks^10, 11^, causing a dominant descending facilitation of nociceptive reflexes and dorsal horn neuron activity in young rats until around postnatal day (P) 28^12–15^. Descending RVM pathways modulate both cutaneous A-fibre and C-fibre sensory inputs to the dorsal horn in young rats^14^, suggesting that there is a postnatal shift in both the direction and modality specificity of descending controls of spinal somatosensation^12, 15^. In the adult, serotonergic raphe-spinal neurons in the RVM are a major source of descending control of nociceptive inputs in the spinal dorsal horn; providing both inhibition and facilitation of pain behaviours and spinal dorsal horn neuron processing of nociceptive inputs^16–19^, most notably in chronic pain states^20^. Strong evidence suggests that descending serotonergic facilitation of nociception is mediated by spinal 5-HT_3_ receptors (5-HT_3_Rs) in chronic pain states but not during acute nociception in adult rodents^21–24^. Serotonergic neurons in the RVM project to the lumbar spinal dorsal horn from birth^25, 26^, but it is not known whether raphe-spinal serotonergic neurons are responsible for the marked functional change in descending modulation of spinal somatosensation over postnatal life.

The aim of this study was to investigate how descending serotonergic neurons modulate dorsal horn neuron processing of cutaneous tactile and noxious mechanical inputs in healthy young rats, and how this descending serotonergic modulation changes with postnatal age. To test this we have measured dorsal horn neuron firing frequency and cutaneous receptive field size, a measure of the excitability of dorsal horn neurons^27^, while pharmacologically manipulating the descending serotonin system at different ages in anaesthetised rats. The results show that descending raphe spinal serotonergic pathways, mediated by 5-HT_3_Rs in the spinal dorsal horn, enhance tactile spinal processing throughout life, but are also responsible for the endogenous facilitation of nociceptive inputs in young animals, before the emergence of a mature balanced descending control.

## Results

### Descending serotonergic fibres facilitate spinal tactile processing throughout life

In the adult, the majority of serotonergic terminals in the spinal dorsal horn arise from cell bodies in the RVM^28^, and these brainstem serotonergic projections to the lumbar spinal dorsal horn are observed from an early postnatal age^25^. To confirm this, we used retrograde tracing to demonstrate that serotonergic neurons in the RVM project to the lumbar spinal cord in young rats and that the proportion of spinally projecting serotonergic RVM neurons increases between P10-P16 (Supplementary Fig. 1A and C). Immunohistochemical staining of 5-HT transporter (5-HTT) to label serotonergic terminals in the lumbar dorsal horn also showed an age-dependent increase in terminal density in the superficial and deep dorsal horn between P7 and P40 (Supplementary Fig. 1E,F).

**Figure 1:**
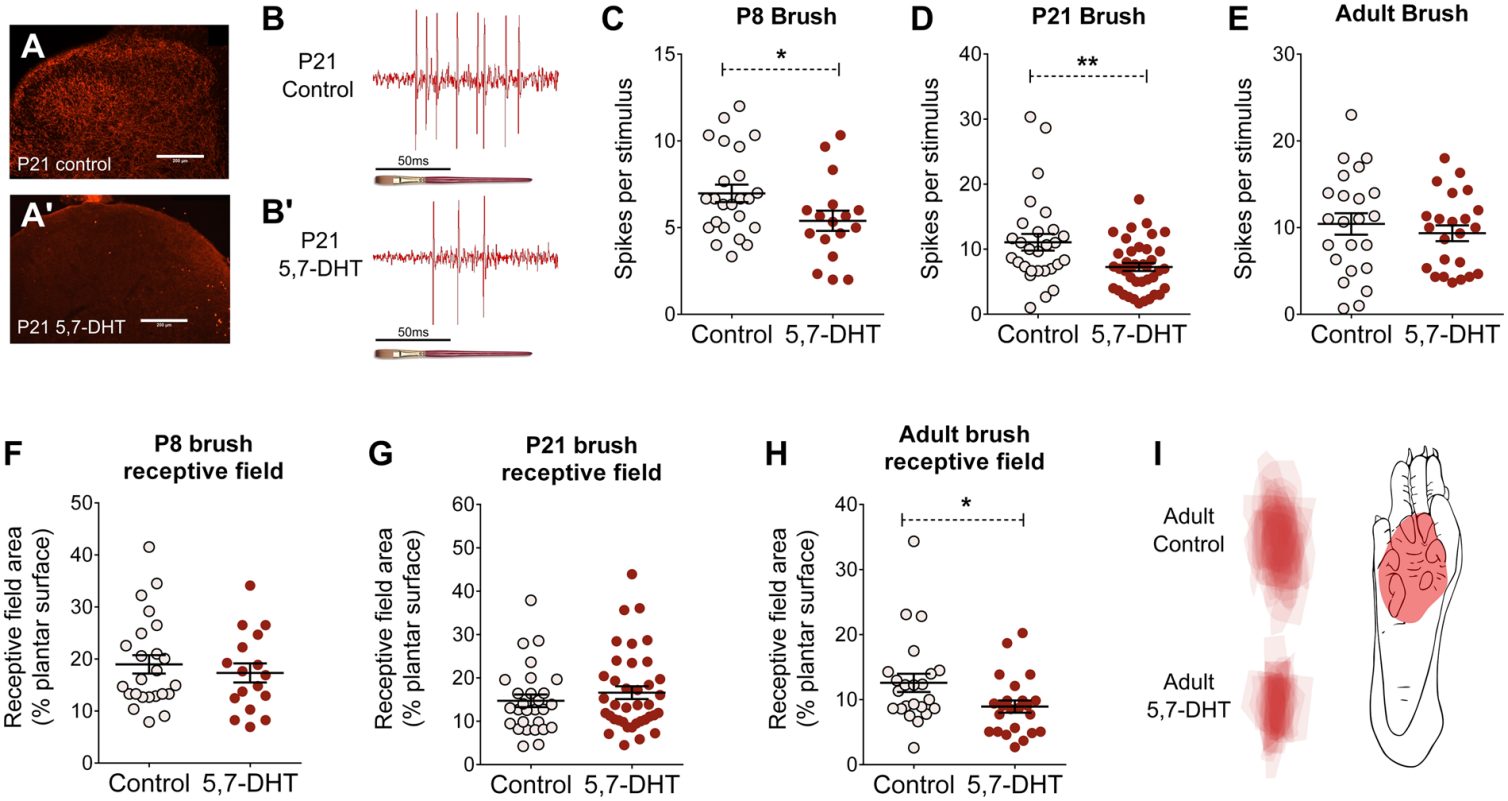
Serotonergic facilitation of dorsal horn neuron brush-evoked activity in young and adult rats. Hindpaw brush-evoked extracellular recordings of wide dynamic range (WDR) neurons were performed in laminae IV-VI of the lumbar spinal dorsal horn at P8, P21 and in adult P45 rats. 5-HT transporter (5-HTT) serotonergic terminals (red) were present in the dorsal horn of saline injected animals (**A**), but not in animals treated with 5,7-DHT (A′). Scale bar = 200 μm. Typical brush-evoked dorsal horn neuron spikes in P21 control rats (**B**) and 5,7-DHT treated rats (B′). Brush-evoked firing activity was lower in 5,7-DHT-treated animals at P8 (**C**) and P21 (**D**), but not in adult rats (**E**). Brush receptive field area was smaller than controls in 5,7-DHT-treated adults (**H**), but not at P8 (**F**) or P21 (**G**). Receptive fields of dorsal horn neurons in control and 5,7-DHT-treated adult groups were overlaid and mapped onto a standardised paw template to create a heat map for each group (**I**). A typical dorsal horn neuron brush receptive field from an adult control rat is shown for scale Bars indicate mean ± SEM. *^,^**P < 0.05, 0.01.

The role of these descending serotonergic pathways on non-noxious, tactile spinal processing in healthy rodents of different ages was investigated using *in vivo* dorsal horn electrophysiology in anaesthetised rats. Spinal cord serotonergic terminals were ablated with intrathecal 5,7-Dihydroxytryptamine, 5,7-DHT (60 µg) injections, 4–5 days before the recording, confirmed by an absence of 5-HT transporter (5-HTT) immunoreactivity in the lumbar spinal cord (Fig. 1A and 1A′). At postnatal day (P)8 (control n = 24 cells; 5,7-DHT n = 17), P21 (control 26; 5,7-DHT n = 39) and adult, P45 (control n = 23; 5,7 = DHT n = 23), cutaneous hindpaw brush-evoked firing activity and receptive field sizes of wide dynamic range (WDR) neurons in the deep laminae of the lumbar dorsal horn were compared in control and serotonin–depleted (5,7-DHT) rats. Typical brush-evoked spike activity recorded from P21 control (Fig. 1B) and 5,7-DHT treated rats (Fig. 1B′) are shown in Fig. 1B.

In young rats, aged postnatal day P8 and P21, mean dorsal horn neuron brush-evoked firing activity was significantly lower in 5,7-DHT-treated compared to control rats (unpaired Student’s t-test, P8 control vs 5,7-DHT, P < 0.05; P21 control vs 5,7-DHT, P < 0.01 Fig. 1C and D), while mean receptive field areas did not differ between the 5,7-DHT-treated and control rats at each age (unpaired Student’s t-test, Fig. 1F and G). In adult rats, mean dorsal horn neuron brush receptive field area was significantly smaller in 5,7-DHT treated rats compared to control (unpaired Student’s t-test, control vs 5,7-DHT, P < 0.05; Fig. 1H), but brush-evoked firing activity did not differ between the two groups (unpaired Student’s t-test; Fig. 1E). Dorsal horn neuron brush receptive field heat maps from adult control and 5,7-DHT-treated rats are shown in Fig. 1I.

These results reveal an endogenous facilitation of low threshold, tactile spinal processing by descending serotonergic fibres, that is present throughout life.

### Descending serotonergic fibres facilitate spinal nociceptive processing in young rats but switch to inhibition in adults

**Figure 2:**
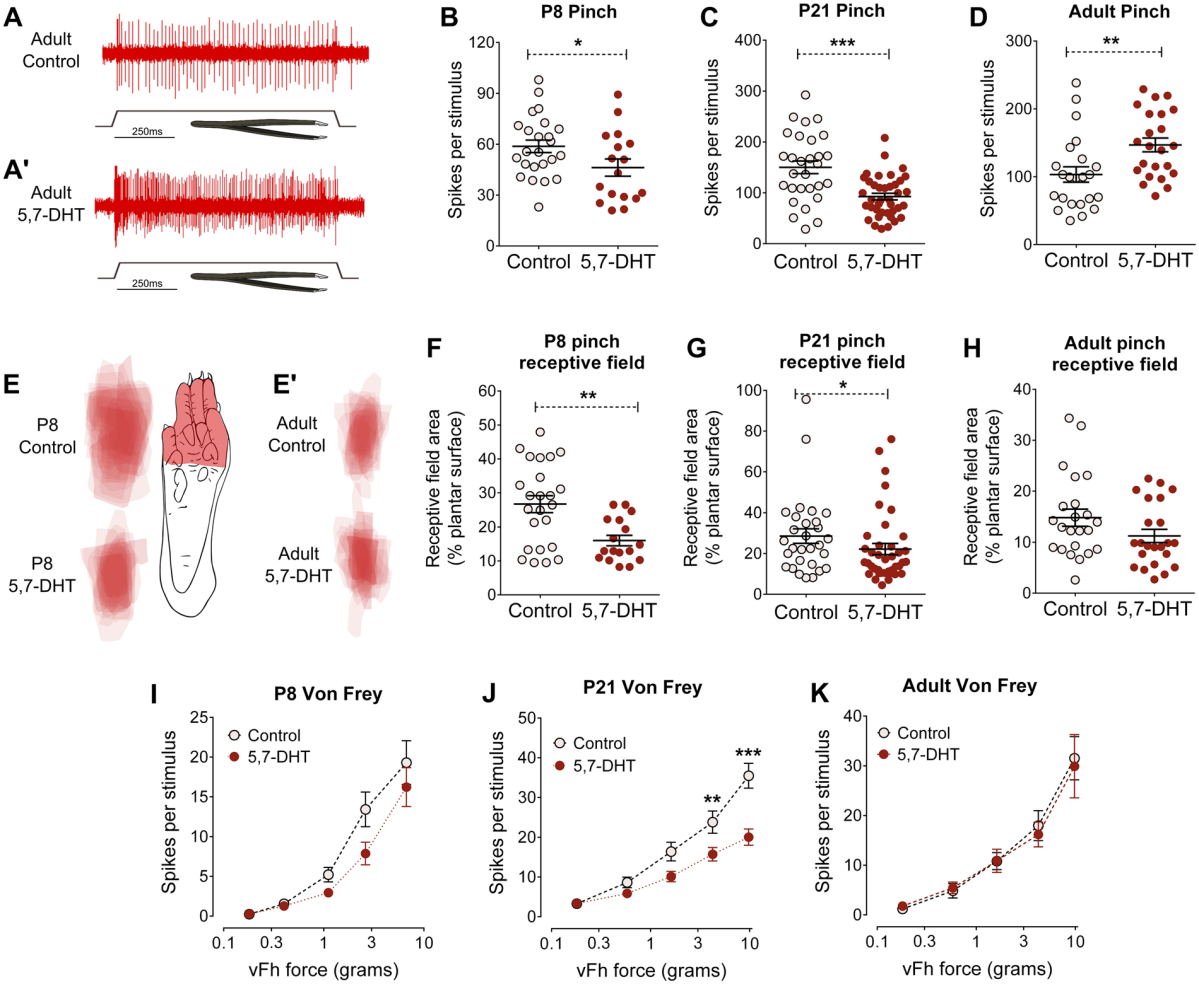
Serotonergic modulation of noxious stimulus-evoked dorsal horn neuron activity is age-dependent. Pinch-evoked activity of dorsal horn WDR neurons was recorded at P8 and P21 and in adult rats. A typical recording of pinch-evoked dorsal horn neuron firing activity in adult control (**A**) and (A′) 5,7- DHT-treated rats. Pinch-evoked firing activity was significantly lower in 5,7-DHT-treated animals compared to controls at P8 (**B**) and P21 (**C**), but higher in adult rats (**D**). Pinch receptive fields were mapped on to a standardised paw template to create heat maps for control and 5,7-DHT-treated rats in P8 (**E**) and adult rats (E′). A typical dorsal horn neuron pinch receptive field from a P8 control rat is shown. Pinch receptive field area was significantly smaller in 5,7-DHT-treated compared to control at P8 (**F**) and P21 (**G**), but not in adult rats (**H**). Dorsal horn neuron responses to increasing forces of von Frey hairs (vFh) was lower in 5,7-DHT-treated compared to controls at P21 (**J**), but not in P8 (**I**), or adult rats (**K**). Bars indicate mean ± SEM. *^,^**^,^***P < 0.05, 0.01, 0.001.

We next investigated the effect of 5,7-DHT treatment on noxious stimulus-evoked firing activity and noxious cutaneous receptive field area on the same group of dorsal horn WDR neurons as described above. At three ages, P8, P21 and adult, P45, cutaneous pinch-evoked firing activity and receptive field sizes and von frey hair-evoked firing activity of WDR neurons in the deep dorsal horn were compared in control and serotonin–depleted (5,7-DHT treated) rats. Typical pinch-evoked firing activity recorded from a dorsal horn neuron in an adult control (Fig. 2A) and 5,7-DHT-treated rat (Fig. 2A′) is shown in Fig. 2A.

At P8, mean pinch-evoked firing activity was significantly lower in 5,7-DHT-treated rats compared to control (unpaired Student’s t-test, control vs 5,7-DHT, P < 0.05; Fig. 2B), and mean pinch receptive field area was significantly smaller in 5,7-DHT-treated rats compared to control rats (unpaired Student’s t-test, control vs 5,7-DHT, P < 0.01 Fig. 2F). Similarly, at P21 mean pinch-evoked firing activity was significantly lower in 5,7-DHT-treated rats compared to control (unpaired Student’s t-test, control vs 5,7-DHT, P < 0.001; Fig. 2C) and mean pinch receptive field area was significantly smaller in 5,7-DHT-treated rats compared to control rats (unpaired Student’s t-test, control vs 5,7-DHT, P < 0.05; Fig. 2G). Additionally, higher force von Frey hair evoked firing activity was significantly lower in 5,7-DHT-treated P21 rats compared to control (Two-way repeated measures ANOVA, control vs. 5,7-DHT, F(1,60) = 5.663, P < 0.01, with Bonferroni post hoc analysis P < 0.01 to P < 0.001 at different forces; Fig. 2J), but not at P8 (Fig. 2I). These results show that high threshold, noxious spinal processing is facilitated by descending serotonergic fibres in young animals.

In contrast, mean pinch-evoked firing activity in adult rats was significantly higher in 5,7-DHT-treated rats compared to control (unpaired Student’s t-test, control vs 5,7-DHT, P < 0.01; Fig. 2A and D), while mean pinch receptive field area did not differ between the two groups (unpaired Student’s t-test; Fig. 2H). Von Frey hair-evoked firing activity did not differ between 5,7-DHT-treated and control adult rats (Fig. 2K). Pinch receptive field heat maps from P8 and adult control and 5,7-DHT-treated rats are shown in Fig. 2E. Note the developmental refinement of dorsal horn neuron receptive fields between P8 and P45 in control groups.

These results show that the well-established descending inhibition of high threshold, noxious spinal processing by descending serotonergic fibres in healthy adults emerges following a switch, from facilitation to inhibition, of descending serotonergic modulation that takes place after P21.

### Spinal 5-HT_3_Rs facilitate dorsal horn tactile processing in both young and adult rats

**Figure 3:**
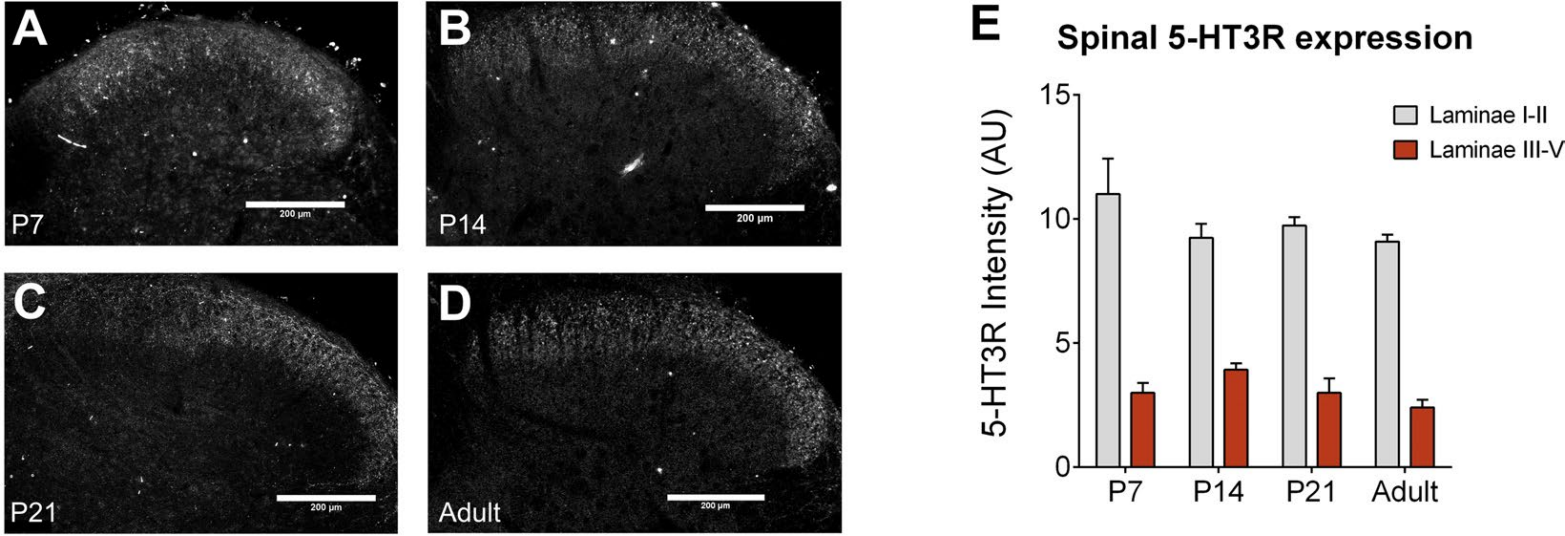
5-HT_3_ receptors are expressed in the lumbar dorsal horn from P7. Immunohistochemical labelling of 5-HT_3_R was performed at P7 (**A**), P14 (**B**), P21 (**C**) and in adults (**D**). Scale bar = 250 μm. 5-HT_3_R labelling was observed predominantly in laminae I-II in the dorsal horn from P7 and this distribution did not change with age. Quantification of 5-HT_3_R intensity was performed in laminae I-II and in laminae III-V and revealed no change in 5-HT_3_R intensity with age (**E**). Bars indicate mean ± SEM.

We next tested whether 5-HT_3_Rs, which are primarily expressed on excitatory dorsal horn interneurons in addition to primary afferent neuron terminals^29, 30^, play a role in serotonergic facilitation of dorsal horn tactile processing. Immunohistochemical experiments demonstrated that 5-HT_3_Rs are expressed in the superficial spinal dorsal horn from P7, and their distribution does not change with age (Fig. 3).

**Figure 4:**
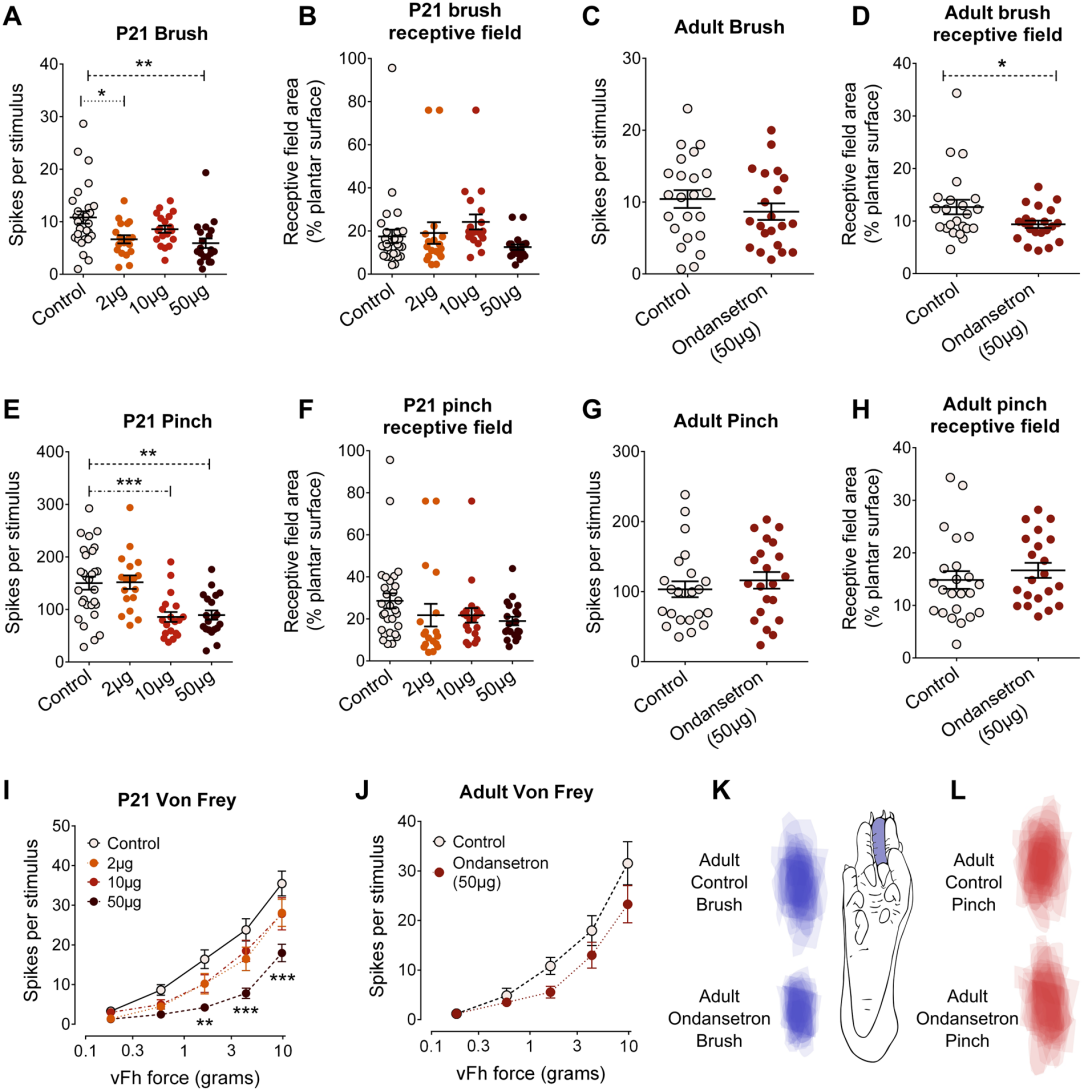
Ondansetron antagonism of spinal 5-HT_3_ receptors has age-dependent effects on dorsal horn neuron activity. The 5-HT_3_R antagonist ondansetron was applied to the surface of the lumbar dorsal horn prior to electrophysiological recordings from WDR neurons at P21 and P40. At P21, 2 μg and 50 μg, but not 10 μg, ondansetron reduced brush-evoked dorsal horn firing activity (**A**), but not receptive field areas (**B**). In adult rats, 50 μg ondansetron reduced brush receptive field area (**D**), but not brush-evoked firing activity (**C**). Pinch-evoked firing activity was reduced in P21 rats following 10 μg or 50 μg ondansetron treatment (**E**). Pinch receptive field areas were not changed (**F**). In adults, 50 μg ondansetron had no effect on pinch evoked firing activity (**G**) or receptive field areas (**H**). 50 μg ondansetron also reduced lower and higher force von Frey hair (vFh)-evoked firing activity in P21 rats (**I**), but not in adults (**J**). Brush (**K**) and pinch (**L**) dorsal horn neuron receptive field heat maps from adult control and ondansetron rats. A typical brush receptive field from an ondansetron-treated adult dorsal horn neuron is shown in (**K**). Bars indicate mean ± SEM. *^,^**^,^***P < 0.05, 0.01, 0.001.

To test the effect of blocking 5-HT_3_Rs in the lumbar dorsal horn on hindpaw brush and pinch-evoked activity of dorsal horn WDR neurons *in vivo*, the 5-HT_3_R-selective antagonist ondansetron was applied to the surface of the lumbar spinal cord of P21 and adult rats and activity was recorded for up to 1 hour after application. At P21, 3 different doses of ondansetron were applied in separate groups of animals (2 µg (n = 18 cells), 10 µg (n = 19) or 50 µg (n = 20) in 50 µl saline) and dorsal horn neuron activity was compared to control animals (n = 26 cells). At P21, mean brush-evoked firing activity was significantly lower in 2 µg and 50 µg ondansetron groups, but not 10 µg, compared to control (One-way ANOVA, control vs 2 µg, 10 µg or 50 µg; F(3,82) = 5.455, P = 0.002; with Dunnett’s post hoc test, 2 µg P < 0.05 and 50 µg P < 0.01 Fig. 4A). There was no difference in brush receptive field area in any drug-treated group compared to control at P21 (n = 26; one-way ANOVA with Dunnett’s *post hoc* test; F(3,82) = 1.827, P = 0.149; Fig. 4B). Additionally, both lower and higher force von Frey hair-evoked firing activity was significantly reduced in animals treated with 50 µg ondansetron (Two-way repeated measures ANOVA, control vs. 50 µg, F(1,42) = 13.01, P < 0.0001, with Bonferroni post hoc analysis, P < 0.01 to P < 0.001 at different forces; Fig. 4I). In adult rats, a single dose of 50 µg ondansetron (in 50 µl saline) was applied to the surface of the lumbar spinal cord. Brush receptive field area of WDR neurons was significantly smaller in ondansetron treated rats (n = 21 cells) compared to control rats (n = 27 cells; unpaired student’s t-test, P < 0.05, Fig. 4D), while brush-evoked firing activity was not changed (unpaired student’s t-test, Fig. 4C).

These results demonstrate an endogenous 5-HT_3_R mediated facilitation of low threshold, tactile activity in the dorsal horn that is present in both young and adult rats.

### Spinal 5-HT_3_Rs facilitate spinal nociceptive processing in young but not adult rats

Since 5-HT_3_R activation facilitates dorsal horn tactile processing in both young and adult animals, we tested their influence on dorsal horn nociceptive processing in the same group of ondansetron-treated rats.

At P21, pinch-evoked firing activity was significantly lower in 10 µg and 50 µg ondansetron groups compared to control (one-way ANOVA, control vs 2 µg, 10 µg or 50 µg; F(3,82) = 9.855, P < 0.0001; with Dunnett’s *post hoc* test, 10 µg and 50 µg P < 0.001 Fig. 4E). Pinch receptive field areas did not differ from control in any group (one-way ANOVA with Dunnett’s *post hoc* test; F(3,82) = 1.397, P = 0.250; Fig. 4F). In contrast, no significant differences between control and ondansetron treated animals were found in pinch-evoked firing activity (unpaired student’s t-test, Fig. 4G), in pinch receptive field area (unpaired student’s t-test, Fig. 4H), or in von Frey hair-evoked firing activity (Fig. 4J) in adult, P45 rats.

These results show an endogenous 5-HT_3_R-mediated facilitation of nociceptive inputs in the dorsal horn of young animals, which is lost in adult animals.

### RVM-spinal serotonergic fibres are the source of descending serotonergic facilitation of dorsal horn nociceptive processing in young rats

In adults, serotonergic descending modulation of spinal processing largely arises from the RVM, which includes brainstem raphe nuclei, although this region is also the source of other non-serotonergic descending fibres^31, 32^. To test whether raphe-spinal serotonergic neurons in the RVM are the source of descending facilitation of nociceptive processing in young P21 rats, we tested whether it was still possible to evoke this facilitation directly with RVM electrical stimulation following serotonergic depletion with 5,7-DHT.

**Figure 5:**
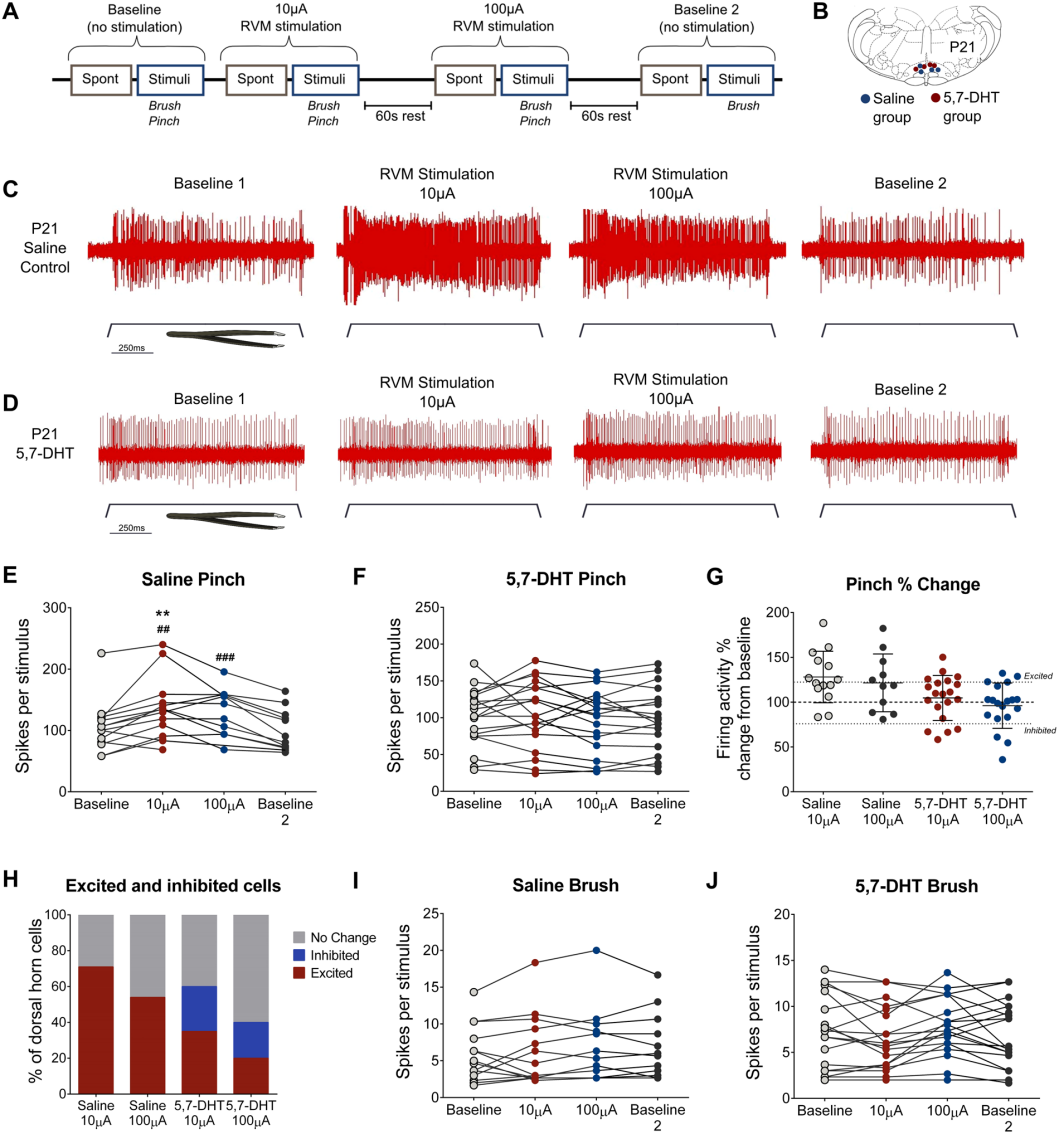
RVM stimulation facilitates dorsal horn neuron activity at P21, but not following ablation of serotonergic terminals. (**A**) Timeline of experiments: brush and pinch-evoked dorsal horn neuron activity was recorded at baseline, during 10 μA stimulation, and during 100 μA stimulation of the RVM. RVM stimulation electrode sites are shown in (**B**). Typical traces of pinch-evoked dorsal horn neuron activity during RVM stimulation bouts are shown in a saline control rat (**C**) and a 5,7-DHT treated rat (**D**). 10 μA and 100 μA RVM stimulation facilitated mean pinch-evoked firing activity in saline-treated rats (**E**), but not 5,7-DHT treated rats (**F**). RVM stimulation increased pinch-evoked firing activity relative to baseline in many individual neurons in saline-treated rats, but not in 5,7-DHT-treated rats (**G**). RVM stimulation increased pinch evoked activity in the majority of cells (>20% firing activity relative to baseline pinch) in saline-treated rats upon (**H**), while in 5,7-DHT treated rats, pinch evoked activity was reduced in a subset of cells (<20% firing activity relative to baseline pinch) by RVM stimulation (**H**). RVM stimulation did not change mean brush-evoked firing activity in saline (**I**) or 5,7-DHT-treated rats (**J**). Bars indicate mean ± SEM. **P < 0.01 versus baseline 1; ^##,###^P < 0.01, 0.001 versus baseline 2.

Dorsal horn neuron firing activity evoked by hindpaw brush and pinch stimulation was recorded in saline (n = 14 cells) or 5,7-DHT treated (n = 20 cells) P21 rats under four conditions: No RVM stimulation (baseline 1); 10 µA RVM stimulation; 100 µA RVM stimulation; no RVM stimulation (baseline 2; Fig. 5A). These stimulation parameters evoke descending excitation (10 µA) and inhibition (100 µA) of dorsal horn neurons and hindlimb or nocifensive tail reflexes in adult rats^12, 33^.

Concurrent with previous findings^14^, RVM stimulation at 10 µA or 100 µA facilitated nociceptive dorsal horn activity in P21 saline treated rats. A typical recording of pinch-evoked firing activity from a dorsal horn neuron in a P21 control rat is shown in Fig. 5C. Mean pinch-evoked firing was significantly higher during 10 µA RVM stimulation when compared to baseline 1 and 2 (paired t-test, baseline 1 or 2 vs. 10 µA, P < 0.01; Fig. 5E) and during 100 µA stimulation when compared to baseline 2 (paired t-test, baseline 1 or 2 vs. 100 µA, P < 0.001; Fig. 5E). When expressed as a percentage change from baseline 1, the majority of cells in saline-treated animals displayed an increase in pinch-evoked firing activity during stimulation (Fig. 5G). A cell with >20% increase in pinch-evoked firing activity during RVM stimulation was defined as excited; the majority of cells were excited during RVM stimulation and no cells were inhibited (Fig. 5H).

In contrast, electrical stimulation of the RVM in 5,7-DHT treated rats had no effect upon mean pinch-evoked firing activity compared to baseline 1 or baseline 2 (One-way repeated measures ANOVA with Bonferroni post hoc comparison, baseline vs. 10 µA vs. 100 µA vs. baseline 2; F = (2.119, 28.15) = 0.763, P = 0.480; Fig. 5F). A typical recording of pinch-evoked firing activity from a dorsal horn neuron in a 5,7-DHT-treated P21 rat is shown in Fig. 5D. When expressed as a percentage change from baseline 1, the majority of cells displayed changes in pinch-evoked firing activity of <20% of baseline activity during RVM stimulation (Fig. 5G), therefore the majority of cells displayed no change in firing activity (Fig. 5H). Additionally, in contrast to controls, RVM electrical stimulation inhibited pinch-evoked firing activity in some cells in 5,7-DHT treated rats (Fig. 5H). Direct RVM stimulation had no effect upon mean low threshold, brush evoked activity in either group (Fig. 5I and J).

These results demonstrate that RVM stimulation-evoked facilitation of nociceptive inputs in the spinal dorsal horn at P21 is highly dependent upon serotonergic signalling, suggesting that descending raphe spinal serotonergic neurons are the major source of descending RVM facilitation of spinal nociception in young rats.

### Age and treatment interactions

As von Frey hair stimuli are the most standardised stimuli across the different ages, we performed three-way ANOVAs to test the effect of age and drug treatment on dorsal horn neuron firing evoked by five vFh forces. Both age (P8, P21 and adult) and 5,7-DHT treatment (control and 5,7-DHT) had strong effects on vFh-evoked firing (repeated measures 3-way ANOVA: F(2,630) 24.116, P < 0.001; F(1,630) = 6.591, P < 0.05, respectively; Supplementary Fig. 2A). Post-hoc t-test analysis, performed on vFh data pooled across all five forces, showed that vFh-evoked firing activity changes significantly with age (Adult vs P21 P = 0.081; Adult vs P8 P = 0.052; P21 vs P8 P < 0.001) and there is a significant interaction of age & treatment F(2,630) = 6.438, P,0.01; Supplementary Fig. 2B.

Three-way ANOVAs also showed a significant effect of ondansetron treatment (50 µg dose only) on vFh evoked firing, (repeated measures 3-way ANOVA: F(1,395) = 7.969, P < 0.01, Supplementary Fig. 2C), and a significant interaction of age and treatment in pooled data (F(1,395) = 28.888, P < 0.001; Supplementary Fig. 2D).

## Discussion

In this study we provide evidence that the source of early life descending facilitation of spinal mechanosensory processing is serotonergic, from raphe-spinal neurons acting upon 5HT_3_Rs in the dorsal horn. Following intrathecal administration of 5,7-DHT to ablate descending serotonergic fibres and deplete 5-HT in the spinal cord, brush and pinch-evoked firing activity of deep dorsal horn neurons was reduced at P8 and P21 compared to age-matched control rats. Antagonism of spinal 5-HT_3_Rs by local application of ondansetron reproduced these results, suggesting that descending facilitation from raphe-spinal serotonergic neurons of low and high-threshold mechanical inputs is mediated by dorsal horn 5-HT_3_Rs in young animals. This serotonergic modulation of nociceptive inputs changes between P21 and P40, when descending serotonergic fibres begin to inhibit, and 5-HT_3_Rs no longer tonically facilitate, nociceptive dorsal horn activity in healthy adult rats. Our results also reveal that the majority of RVM facilitation of nociceptive processing in the spinal dorsal horn in young P21 rats arises from descending RVM serotonergic neurons.

### Descending Serotonergic Inhibition of Spinal Nociception in Adulthood

Descending modulation of nociceptive processing in the dorsal horn from the brainstem is well established in laboratory rodent and human studies^3, 34–37^ and arises in part from both serotonergic and non-serotonergic neuron populations in the RVM^7, 31^. In the adult, descending modulation of spinal nociception arising from the brainstem is balanced, either facilitating or inhibiting nociceptive activity, depending on the behavioural state of the animal in different environments^38–40^. Descending brainstem pathways also have a degree of functional selectivity, as descending modulation from the periaqueductal grey, PAG and rostroventral medulla, RVM selectively dampens the activity of wide dynamic range neurons with nociceptive inputs from C-fibres^6, 14, 41^.

Recent research has focussed on role of the serotonergic system in pain states^42^, but descending serotonergic controls also modulate processing of spinal nociception and pain behaviours in uninjured, healthy animals. Under these conditions, an antinociceptive role of descending serotonergic modulation was reported over thirty years ago^43, 44^. The results reported here provide additional evidence of serotonergic inhibition of nociceptive processing in the dorsal horn of uninjured adults rats that was not mediated by spinal 5-HT_3_Rs, and therefore involve other serotonergic receptor subtypes, such as 5HT_1A_R^45^. There is evidence of descending facilitation of pain behaviour^19^ and bladder sensitivity^46^ in adult healthy rats, and 5,7-DHT injection has been reported to reduce noxious mechanical and heat-evoked dorsal horn WDR neuron firing activity^17^, perhaps due to selection of different WDR populations from those recorded here. These seemingly contradictory findings support a hypothesis that, in adult animals, descending facilitatory and inhibitory somatosensory controls operate in parallel to produce a modulatory outcome which is appropriate to the behavioural state of the animal in different environments^39, 47^.

The mechanism by which serotonergic inhibitory controls of nociceptive processing mature in the adult spinal cord are not known. Sensorimotor outcomes of serotonin release in the spinal dorsal horn depend in part upon which serotonin receptor subtype activated^48^. For example, activation of 5-HT_7_Rs causes inhibition of pain behaviours in pain states^22^, and it is possible that developmental changes in serotonin receptor subtypes may underlie postnatal maturation of serotonergic inhibition at the level of the spinal cord. 5HT-R are also expressed by astrocytes, which can modulate nociceptive circuits^49^ in a developmentally regulated manner^50^. In addition, developmental changes in excitatory-inhibitory transmission in spinal dorsal horn sensory circuits during the first weeks of life may underlie changes in descending brainstem controls^51–53^.

### Descending Serotonergic Facilitation in Young and Adult Life

Here, we present novel evidence of descending serotonergic facilitation of processing of low-threshold tactile inputs in the dorsal horn of rats of all ages. The serotonergic/5-HT_3_R-mediated facilitation of tactile inputs in the dorsal horn of young rats is still present in adults and therefore appears to be present throughout life. This is consistent with reports from the 1980s showing that descending PAG-RVM controls of spinal somatosensation are not nociceptive-selective and also target non-noxious inputs^8, 9^; evidence that has been largely overlooked in recent studies. Serotonergic facilitation of tactile inputs has not been reported elsewhere, although inspection of the data in Rahman *et al*.^17^ shows higher dorsal horn neuron firing activity in 5,7-DHT animals compared to control animals in response to lower force von Frey hairs (Rahman *et al*.^17^).

We show that serotonergic facilitation of tactile inputs is mediated by 5-HT_3_Rs in the dorsal horn. In the adult, the majority of 5-HT_3_R expression is on excitatory VGLUT2+ interneurons in the superficial spinal dorsal horn ^29^, and it is possible that 5-HT_3_R-mediated facilitation of low-threshold inputs is mediated by 5-HT_3_R-expressing excitatory interneuron populations which receive low-threshold sensory inputs. Possible candidates include somatostatin, VGLUT3, RORα, or PKCγ excitatory interneuron populations which receive monosynaptic Aβ inputs and provide feedforward excitation of projection neurons^53–55^. The ability of descending serotonergic fibres to modulate spinal cord circuits driven by both non-noxious and noxious sensory inputs provide a powerful mechanism for the brain to alter the gain of both tactile and nociceptive processing in the spinal cord.

### Selectivity of Serotonergic Control

Evidence of serotonergic neuromodulation of sensory neuron responsiveness and selectivity to synaptic inputs is well established in mammalian brainstem and cortical sensory networks (Hurley *et al*., 2004). By altering the signal-to-noise ratio of neurons in the sensorimotor cortex (Foehring *et al*., 2002) or changing visual evoked response thresholds and receptive field sizes of neurons in the primary visual cortex (Waterhouse *et al*., 1990), serotonin release can dramatically alter how sensory inputs are processed in primary sensory networks. Thus, serotoninergic controls can dynamically act not just to alter the gain of sensory processing, but also to alter stimulus coding in sensory networks.

**Figure 6:**
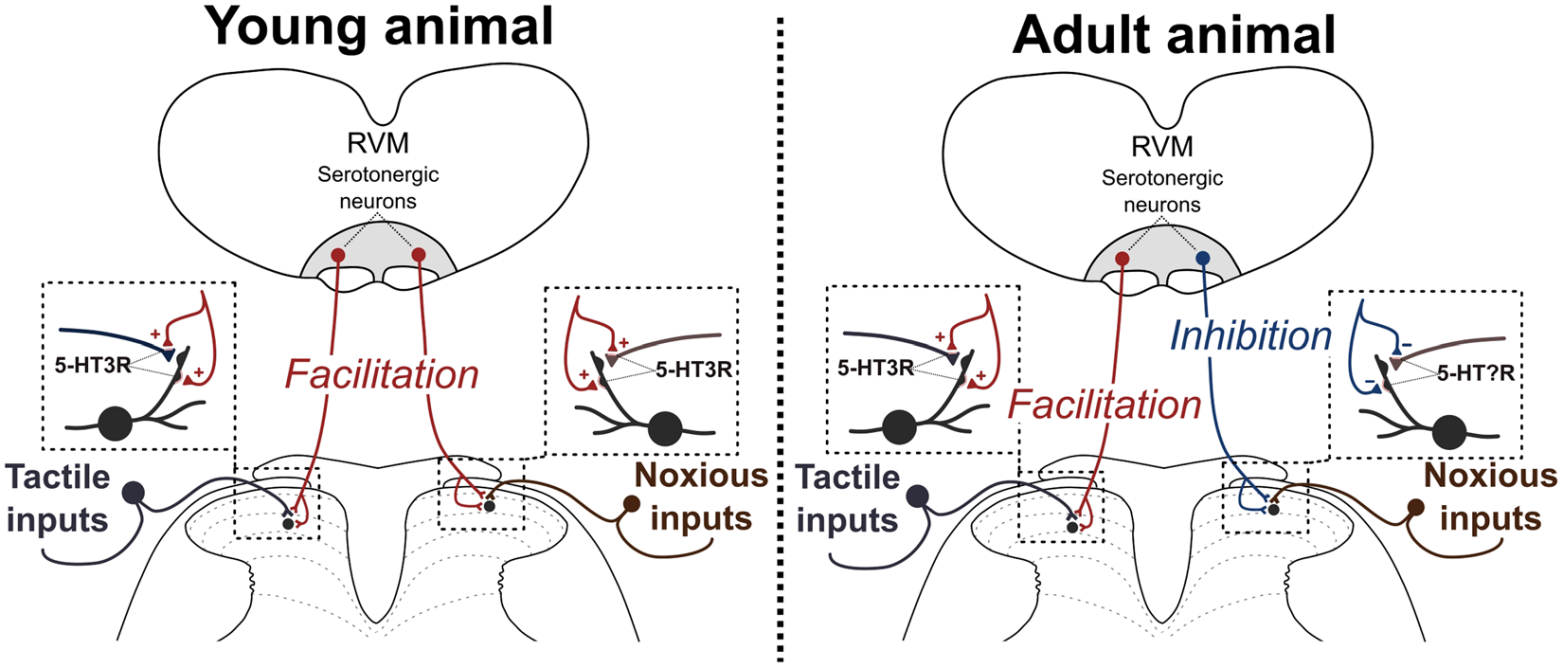
A proposed model of serotonergic descending modulation of spinal somatosensation. In young animals (P21), descending serotonergic modulation facilitates tactile and noxious inputs to the dorsal horn via activation of 5-HT_3_Rs. In adults, descending serotonergic and 5-HT_3_R-mediated facilitation of tactile inputs is also observed; however, noxious inputs are now inhibited by descending serotonergic neurons via a different 5-HTR subtype.

Here, we demonstrate that the selectivity of serotonergic control of spinal somatosensation changes with postnatal age. In young animals, descending serotonergic control is non-selective and amplifies the saliency of low and high-threshold mechanical sensory inputs in the spinal cord; both by increasing neuronal activity and spatial receptive field sizes of dorsal horn neurons. Importantly, we show that this non-selective serotonergic control was mediated by spinal 5-HT_3_Rs in young rats (Fig. 6). In contrast, serotonergic controls have a more balanced and nuanced role in the adult by either increasing or decreasing the relative saliency of low or high-threshold mechanosensory inputs in the dorsal horn (Fig. 6). These findings support and add new insight into previous reports that balanced descending excitatory and inhibitory control of spinal nociception from the brainstem is not established at birth, and endogenous RVM modulation of spinal nociception undergoes considerable postnatal change between P21-P40^12, 15, 56^ under central opioidergic control^13, 56^.

### A Role for Serotonergic Facilitation in Early Life

Descending facilitatory drive in the first postnatal weeks may be an important source of excitation which acts to reinforce and strengthen functionally relevant synapses in the dorsal horn. Serotonergic transmission in the neonatal spinal cord is known to promote maturation of sensory-motor circuits: in rat P2-17 spinal cord slices, 5-HT enhances dorsal horn neuron EPSCs by accelerating the maturation of silent glutamatergic synapses (Li and Zhuo, 1998b). In the neonatal spinal ventral horn, serotonergic transmission increases the excitability of intersegmental commissural interneurons at P14-16 (Abbinanti *et al*., 2012) and promotes plasticity of GABAergic transmission (Sadlaoud *et al*., 2010). It is therefore possible that early descending serotonergic facilitation may be involved in the maturation of both excitatory and inhibitory transmission in the dorsal horn.

Our results suggest that descending facilitation from the brainstem arises from serotonergic neurons in the RVM in young rats (Fig. 5). Moreover, as ablating these descending serotonergic fibres unmasked dorsal horn neurons which were inhibited upon electrical stimulation of the RVM, we propose that RVM serotonergic neurons normally override weaker descending RVM inhibitory controls which likely involve non-serotonergic neurons. We hypothesise that serotonergic controls may mask maturing descending inhibition from the RVM to promote activity-dependent maturation of spinal sensory processing in the first postnatal weeks.

## Conclusion

Collectively, these findings identify serotonergic/5-HT_3_R signalling as an important mechanism which mediates dominant descending RVM facilitation of both low and high thresholds sensory inputs to the dorsal horn during early life and continues to facilitate tactile inputs in adults.

## Materials and Methods

### Animals

All experiments were performed in accordance with the United Kingdom Animal (Scientific Procedures) Act 1986 with approval from the United Kingdom Home Office. Reporting is based on the ARRIVE Guidelines for Reporting Animal Research developed by the National Centre for Replacement, Refinement and Reduction of Animals in Research, London, United Kingdom^57^. Male and female Sprague-Dawley rats were obtained from the Biological Services Unit, University College London. Rats were bred and maintained in-house and exposed to the same caging, diet and handling throughout development. Litters were weaned at P21 into same sex cages of four littermates and were housed in 12 h light/dark cycles at constant ambient temperature and humidity with free access to water and food.

### Drugs

For descending serotonergic neuron ablation experiments, 5,7-di-hydroxytryptamine creatine sulphate (5,7-DHT) (Sigma-Aldrich, UK) was dissolved in saline to a concentration of 3 mg/ml. To prevent noradrenergic neuron toxicity, rats were pre-treated with Desipramine (Sigma-Aldrich) dissolved in saline at a concentration of 5 mg/ml. Desipramine (25 mg/kg) was intraperitoneally (I.P) injected 1 hour before 5,7-DHT or saline injection. For intrathecal injections, rats were deeply anaesthetised with isoflurane and low lumbar spinous processes were visualised through a small midline skin incision. A 30-gauge needle was passed in the midline through the lumbar (L) 4/5 or L5/6 intervertebral space to perform intrathecal injection. The same injectate concentration and volume of 5,7-DHT (60 µg in 20 µl) was used in all ages to ensure sufficient spread of injectate to the lumbar 4/5 spinal cord in animals of all ages^58^. Control animals received equivalent volumes of saline. The skin overlying the injection site was then sutured with 5–0 suture (Ethicon), EMLA cream (AstraZeneca) was placed on the wound, and animals were returned to the mothers or back to home cages for five days before electrophysiology experiments.

In 5-HT_3_R antagonism experiments, the selective 5-HT_3_R antagonist Ondansetron Hydrochloride (Tocris Biosciences, UK) was dissolved in saline to a concentration of 0.4 µg/µl, 2 µg/µl or 10 µg/µl. A 50 µl solution containing 2 µg, 10 µg or 50 µg of Ondansetron was then applied to the surface of the L4/5 spinal dorsal horn during electrophysiology experiments and dorsal horn neuron activity was recorded for up to 1 hour to ensure recordings were performed during the period of maximum effect of ondansetron (Rahman *et al*.^59^).

### *In vivo* extracellular recordings in the dorsal horn preparation

Rats were anaesthetised with isoflurane (induction 4% in medical O_2_, Univentor Anaesthesia Unit 400; Royem Scientific, UK), tracheotomised and artificially ventilated under constant isoflurane anaesthesia (maintenance of 1.8%). The air flow and breathing rate were adjusted to the animal’s sizes using a small animal ventilator (model 687, Harvard Apparatus, MA, USA). Heart rate was constantly monitored via electrocardiogram and a homoeothermic blanket with feedback control (model 507220 F, Harvard Apparatus, MA, USA) was used to maintain body temperature at physiological levels. The rat was mounted onto a stereotaxic frame (Kopf Instruments, Tujunga, CA, USA). A laminectomy was performed to expose the lumbar spinal cord, the vertebral column was secured with a clamp to the thoracic site and the dura and pia mater were removed. A film of mineral oil was used to cover the exposed spinal cord to prevent heat loss. The skull was exposed and bregma located to perform a small craniotomy for unilateral RVM microinjection. Stereotaxic coordinates for the P21 RVM were calculated as outlined previously^12^: lateral 0 mm, antero-posterior 9.2 m, dorso-ventral −10.0 mm.

#### Neuronal Recording

To isolate individual neurons in the dorsal horn, a 6 µm tipped glass-coated carbon fibre microelectrode (Kation Scientific, Minneapolis, USA) was lowered through the spinal cord with an *in vivo* manipulator (Scientifica, UK) while stroking the plantar surface of the hindpaw as a search stimulus for dorsal horn wide dynamic range (WDR) neurons in lamina IV-VI. All recorded WDR neurons had receptive fields in the glabrous skin. Mean recording depth at P8 was 405.4 µm, at P21 was 529.7 µm and at P40 was 577.4 µm, reflecting the growth of the spinal cord during postnatal development. Cutaneous glabrous receptive fields to brush and pinch stimulation were mapped and the number of spikes per stimulus to brush, pinch and von Frey hair (vFh) stimulation of the receptive field were recorded. The brush stimulus used was a fine acrylic paintbrush with a 1 mm tip, applied across the hindpaw receptive field over 0.5 s. Pinch stimulation in all ages was performed with a pair of F.S.T. curved serrated forceps (product code 11152-10) with a 0.3 mm tip. The centre of the receptive field was pinched until the arms of the forceps just began to bend, and was applied for 2 seconds, providing consistent pinch stimuli. Von Frey hairs ranging from 0.18 g to 9.80 g were applied to the centre of the hindpaw receptive field for 1 second. To avoid overstimulation and sensitisation of sensory afferents, the maximum vFh forced applied to P8 rats was 6.70 g, and to P21 and adult rats was 9.80 g. Spontaneous neuronal activity was recorded for one minute before and after hindpaw stimulation protocols. Stimulus evoked action potentials were digitalised using PowerLab 4/30 interface and isolated using the Chart 5 software spike histogram plug-in (AD Instruments Ltd, Oxford, UK). Animals were sacrificed and perfused immediately after recordings to process tissue for immunohistochemistry.

### RVM Microstimulation

In RVM microstimulation experiments in P21 rats, dorsal horn wide dynamic range (WDR) neuron baseline brush, pinch and von Frey hair receptive fields and firing activity were initially characterised in the absence of electrical stimulation for baseline recordings. Then, trains of electrical stimuli of 500 µs pulse width were applied at 10 Hz, at 10 and 100 µA using a stimulus isolator (NeuroLog). A second set of WDR neuron baseline firing properties was performed after electrical stimulation (see Fig. 5 for summary). These stimulation parameters reliable evoke descending excitation (10 µA) and inhibition (100 µA) of dorsal horn neuron and hindlimb or tail reflex activities in adult rats^12, 14, 33^. At the end of the experiment animals were transcardially perfused for tissue processing and immunohistochemistry. To inspect stimulation sites, the cerebellum was removed, and the brainstem was cut coronally with a scalpel blade to allow visual inspection of the tract mark into the RVM.

### Spinal cord immunohistochemistry

For spinal cord 5-HT_3_R immunohistochemistry experiments, naïve P7, P14, P21 and P40 rats (n = 4 per age) were perfused and lumbar spinal cord tissue was harvested. Tyramide signal amplification (TSA) immunostaining was performed on spinal cord sections. Free floating sections were blocked with 3% goat serum in 0.3% Triton X-100 in 0.1 M PBS for 1 h at room temperature (RT). Spinal cord sections were then incubated overnight at room temperature with 5-HT_3_R antibody (rabbit, 1:1000, Millipore). The next day, sections were incubated in biotinylated anti-rabbit antibody (goat anti-rabbit; 1:400; Vector Stain) for 90 mins. Sections were washed thrice in 0.1 M PBS over 30 mins, then placed in ABC complex (1:125; Vector Stain, ABC elite kit, Vector Labs) for 30 mins. Sections were washed thrice over 30 mins and then placed in biotinylated tyramide (1:75; TSA Stain Kit; Perkin Elmer) for 7 mins. Sections were then washed thrice and incubated in fluorescence Isothiocyanate (FITC; 1:600; Vector Stain) for 2 hours. Sections were washed thrice and mounted on gelatinised slides and cover slipped with Fluoromount (Sigma).

For 5-HT_3_R quantification, mean fluorescence intensity was measured in two 100 µm × 100 µm ROIs; one ROI in the superficial dorsal horn (laminae I and II), and one ROI in the deep dorsal horn (laminae III-V). The mean intensity of 5-HT_3_R for each sub-region of interest was measured using ImageJ/Fiji image analysis software. The intensities of 4–5 sections per animal were averaged to create one *n* value per animal.

### Statistical analyses

Statistical analyses and graphing were performed using GraphPad Prism 6 (GraphPad software, La Jolla, CA, USA) and *P* < 0.05 was considered statistically significant. Sample sizes for testing were based on previously reported group differences between RVM silenced/stimulated animals in electrophysiology experiments^5, 14, 60^. Evoked cell response values are expressed as the mean brush, pinch or vFh-evoked firing activity of three stimuli. Dorsal horn neuron receptive fields were drawn on a standardised template during recording and then imported into Inkscape (version 0.48 www.inkscape.org) where pixel count was used to measure the area. Receptive fields are expressed as a percentage of the total area of the hindpaw plantar surface. Receptive fields were normalised to the standardised paw template and overlaid and centred to create receptive field heat maps for each group. A 10% opacity level was used for each receptive field to create the heat maps.

In all except the RVM stimulation experiments, population-based statistical comparisons were performed. Dorsal horn neuron recordings from 5,7-DHT-treated or ondansetron-treated animals were pooled and treated as one population of neurons for each age. The control cell population is a pooled group of cells from animals receiving RVM saline (P8 = 7 cells form 2 animals; P21 = 15 cells from 2 animals; adult (13 cells from 2 animal) and naïve animals which displayed the same cell properties. In normally distributed data sets, group differences between drug treated (5,7-DHT or ondansetron) and between control animals within age groups were tested with unpaired Student’s t-tests or one-way analysis of variance (ANOVA) followed by Bonferroni *post hoc* multiple comparisons tests. Data sets which were not normally distributed were compared with Mann-Whitney or Kruskal-Wallis tests. In von Frey hair experiments, Two-way repeated measures ANOVA were used within the age groups followed by Bonferroni *post hoc* multiple comparison test to compare differences in responses to increasing von Frey hair force in control animals and drug-treated animals.

In experiments with RVM electrical stimulation in P21 rats, within subject repeated measures statistical comparisons were used. The effect of RVM stimulation at different amplitudes (10 µA and 100 µA) on WDR neuron firing and receptive field properties were compared to baseline recordings from the same cell. Brush and pinch-evoked firing activities were compared to two baselines; one before and one after electrical stimulation bouts to ensure changes in WDR neuron firing properties were not long term after RVM stimulation and repeated hindpaw stimulation. All other comparisons were compared to baseline conditions before electrical stimulation bouts. Repeated measures one-way or two-way ANOVAs with Bonferroni *post hoc* analysis were performed to analyse within-cell and between population (baseline vs 10 µA or 100 µA) changes caused by RVM stimulation. Data sets which were not normally distributed were compared with repeated measures Friedman Tests. Brush and pinch-evoked firing activity data from saline and 5,7-DHT treated animals was also expressed as the percentage change from baseline 1 firing activity. Cells were classified as facilitated or inhibited by RVM stimulation if firing rates increased or decreased by >20% of baseline values, respectively. This threshold was chosen as it is above the normal level of variability observed between the two baseline recordings.

## Electronic supplementary material

Supplementary Information
