# Supplementary material for: The developmental emergence of differential brainstem serotonergic control of the sensory spinal cord
Source: Sci Rep. 2017 May 22;7:2215. doi: 10.1038/s41598-017-02509-2 (PMC5440407; doi:10.1038/s41598-017-02509-2)
Supplement: Supplementary file 1 — Supplementary Information [file 41598_2017_2509_MOESM1_ESM.pdf]

The developmental emergence of differential brainstem serotonergic control of the sensory spinal cord

Schwaller F*[1,2,], Kanellopoulos AH[1], Fitzgerald M*[1]

Supplementary data

Supplementary Figure 1

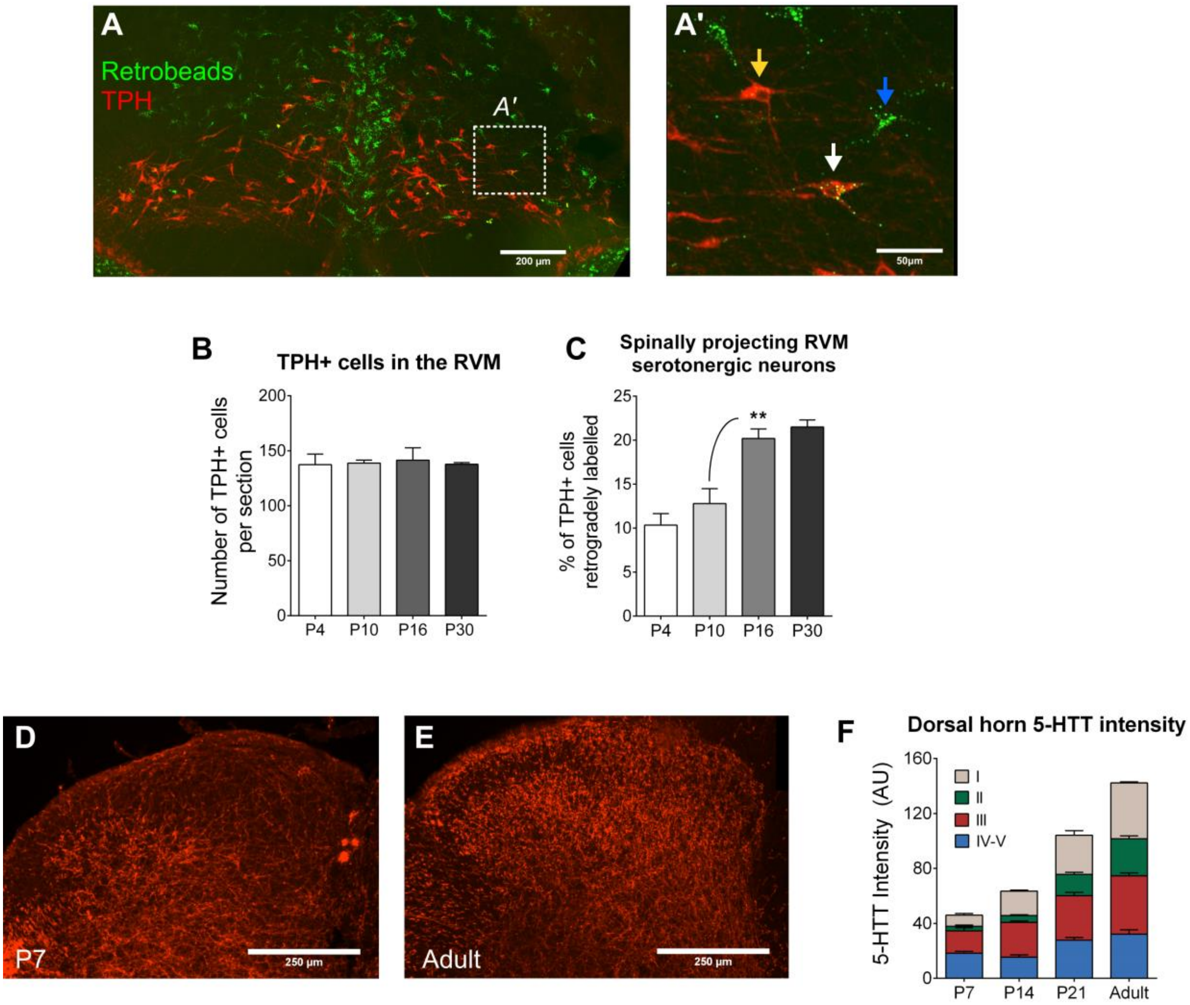

Supplementary figure 1. Rostroventral medulla serotonergic neuron innervation of the lumbar spinal dorsal horn

(**A**) Brainstem RVM section from a P4 rat injected with Retrobeads into the lumbar dorsal horn at birth. Scale bar = 250µm. **(A')** Excerpt from **(A)**; Retrobead-containing cell bodies are green (blue arrow), serotonergic tryptophan hydroxylase (TPH+) cell bodies are red (yellow arrow; co-labelled cell = white arrow). Scale bar = 50µm. Quantification of the average number of TPH+ cells in the RVM demonstrated no change in the number of serotonergic neurons between P4 and P30 (**B**). The proportion of serotonergic neurons which project to the lumbar dorsal horn increased between P10 and P16 **(C)**. Lumbar spinal cord sections were stained with 5-HT transporter (5-HTT) to label serotonergic terminals: example images from P7 **(D)** and adult P40 **(E)** rats are shown. Scale bar = 250µm. Quantification of 5-HTT intensity in different dorsal horn laminae at different ages demonstrated 5-HTT intensity increases in different laminae and throughout the dorsal horn with age **(F)**. Bars = mean ± SEM. ** P<0.01

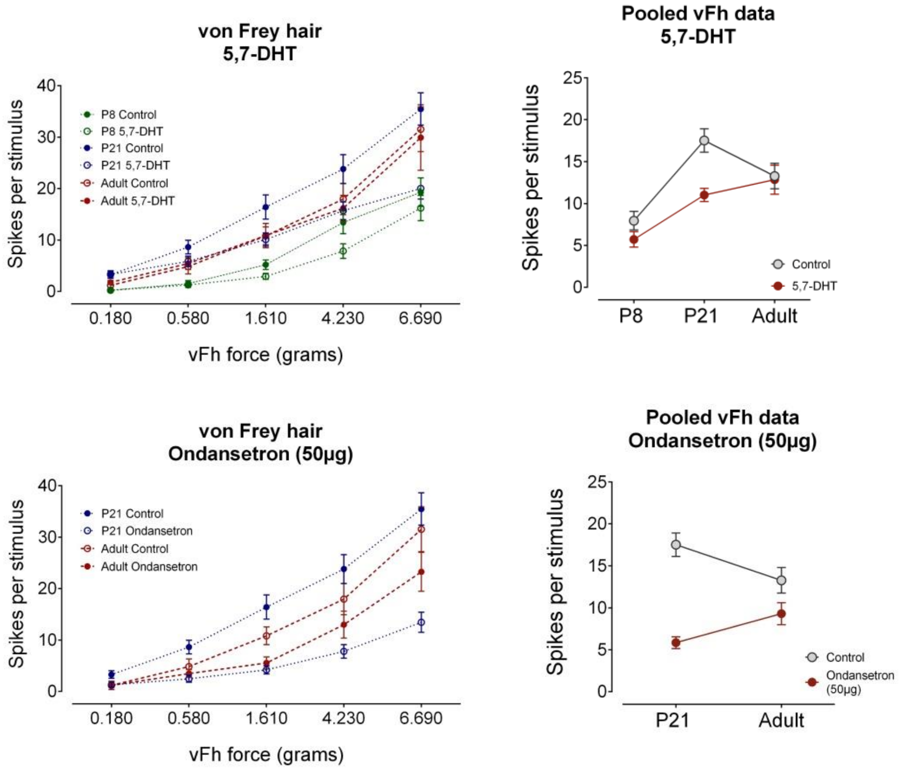

Supplementary figure 2. Age and treatment interactions on vFh-evoked firing activity (**A**) Graph showing the effects of 5,7-DHT treatment on vFh-evoked firing activity at three ages, P8, P21 and adult. Three-way ANOVA revealed significant effects of age and 5,7-DHT treatment on vFh-evoked firing activity and (B) a significant age-treatment interaction when all five vFh data points are pooled. (**C**) Graph showing the effects of odansetron treatment (50µg) on vFh-evoked firing activity at two ages, P21 and adult. Three-way ANOVA revealed significant effects of odensatron treatment on vFh-evoked firing activity and (D) a significant age-treatment interaction when all five vFh data points are pooled (see text for details). Bars = mean ± SEM.

Supplementary Results

Retrograde tracing and immunohistochemistry

In retrograde tracing experiments, rats aged P0, P6 and P12 (n=4 per age) were anaesthetised with isoflurane (induction 4% in medical O_2, maintenance 2.5%). An incision was made over the lumbar 4-5 spinal dorsal horn and a small bilateral laminectomy was then made. A 500nl injection of microspheres (Retrobeads, Lumafluor) was made directly into the left lumbar 4-5 spinal dorsal horn using a 26 gauge Hamilton syringe. The needle was left in place for 30 seconds before withdrawal. P26 animals received intraspinal injection of 1µl of FluoroGold (Fluorochrome, n=4) instead of Microspheres, due to poor tracing efficacy of Microspheres in adult animals. The muscle and skin overlying the injection site was then sutured with 5-0 suture (Ethicon).

Animals were perfused four days after intraspinal injections of retrograde tracers. Rats were anaesthetised with pentobarbitone sodium (500mg/kg) and transcardially perfused with heparinised saline (5000 IU/ml) followed by 4% paraformaldehyde (PFA) in 0.1 M phosphate buffer. The brain and lumbar spinal cord were removed and postfixed overnight in 4% PFA and transferred to a 30% sucrose solution in 0.1M phosphate buffer solution (PBS) containing 0.01% sodium azide and stored at 4°C. Brain and spinal cord tissue was sectioned on a freezing microtome at 40µm and 30µm thicknesses, respectively. Brainstem sections were blocked with 3% goat serum in 0.3% Triton X-100 in 0.1M PBS for 1h at room temperature, incubated overnight with tryptophan hydroxylase (TPH; mouse, 1:500, Sigma Aldrich), washed thrice with 0.1M PBS, then incubated with goat anti-mouse Alexafluor 594 (1:250).

For spinal 5-HTT immunohistochemistry experiments, naïve P7, P14, P21 and P40 rats (n=4 per age) were perfused and lumbar spinal cord tissue was harvested. Sections were blocked with 3% goat serum in 0.3% Triton X-100 in 0.1M PBS for 1h at room temperature, incubated overnight with 5-HT transporter antibody (5-HTT; rabbit, 1:10000, Immunostar), washed thrice with 0.1M PBS, then incubated with goat anti-mouse Alexafluor 594 (1:250). Spinal cord sections from 5,7-DHT-treated rats were also labelled with 5-HTT to confirm ablation of serotonergic terminals (Fig 1A). All sections were washed after secondary antibody steps and mounted on gelatinised slides and were then cover slipped with Fluoromount (Sigma). Negative control stains omitting primary antibodies resulted in no immunofluorescence, demonstrating no non-specificity of any protocol. Sections were viewed using a Leica DMR light microscope, photographed using a Hamamatsu C4742-95 digital camera and analysed with Volocity Software 6.3.

Immunohistochemistry data collection: In retrograde labelling experiments, TPH+ and Microsphere/Fluorogold & TPH-co-labelled cells were counted in the RVM. Data is expressed as the percentage of TPH+ cells which were retrogradely labelled, and was calculated as the number of Microsphere/FluoroGold & TPH+ cells divided by the number of TPH+ cells. Counts were performed in four sections per animals and were averaged to create one *n* per animal. For 5-HTT quantification, mean intensity was measured in 20µm x 30µm ROIs in lamina I, II, III and IV-V. The mean intensity of 5-HTT for each sub-region of interest was measured using ImageJ/Fiji image analysis software. The intensities of 4-5 sections per animal were averaged to create one *n* value per animal.
